# Supplementary material for: Characterizing the Roles of Life Stage and Season on the Prevalence of Select Viral Pathogens in Acheta domesticus Crickets on a Commercial Cricket Farm in the United States
Source: Vet Sci. 2025 Feb 20;12(3):191. doi: 10.3390/vetsci12030191 (PMC11946654; doi:10.3390/vetsci12030191)
Supplement: Supplementary file 1 [file vetsci-12-00191-s001.zip › Supplementary Figures S1-S4.pdf]

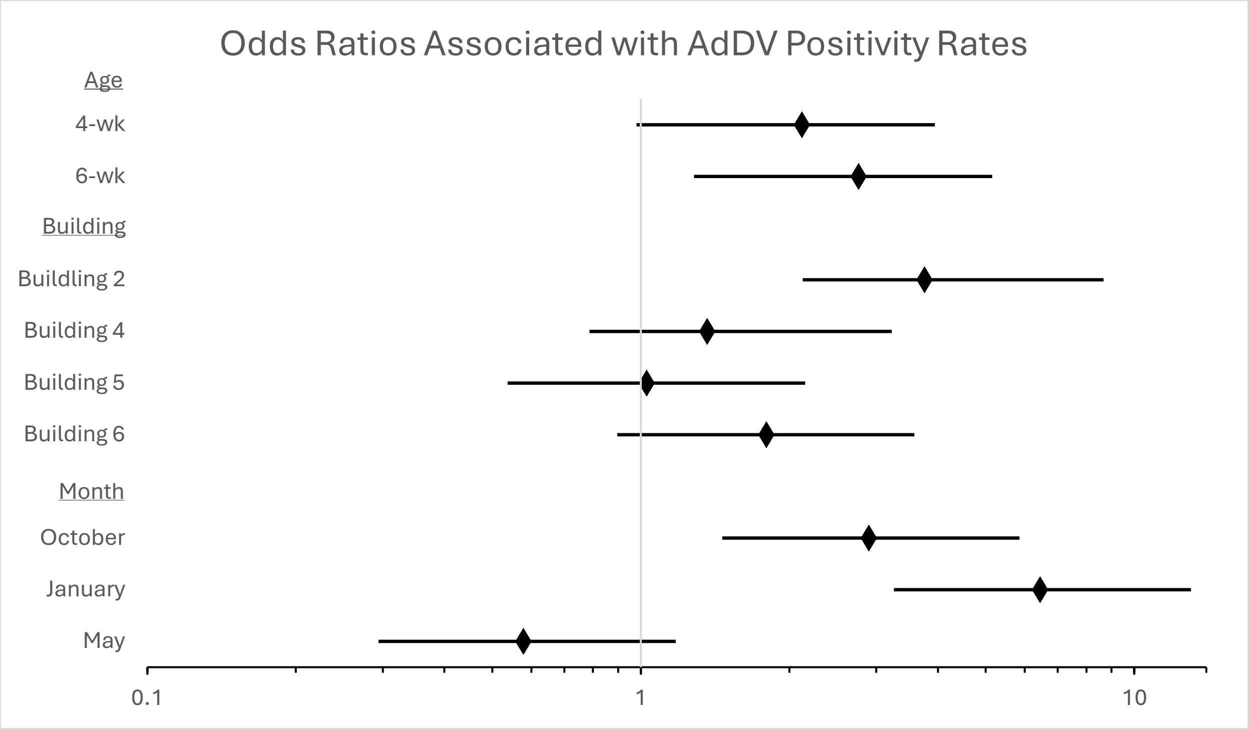

Supplementary Figure S1. This Forest plot graphically displays the odds ratios associated with AdDV positivity rates for each of the risk factors described (age, building, month). For age, 4-week and 6-week crickets were more likely to test positive as compared to the 2-week crickets that were used as the model reference. For building, Building 2, was the most likely building to house positive crickets. Building 1 was used as the model reference and Buildings 4,5, and 6 all had confidence intervals that crossed “1” indicating similar results to Building 1. For month, October and January were more likely to have positive cricket samples, while May and August (the model reference) had similar results to each other.

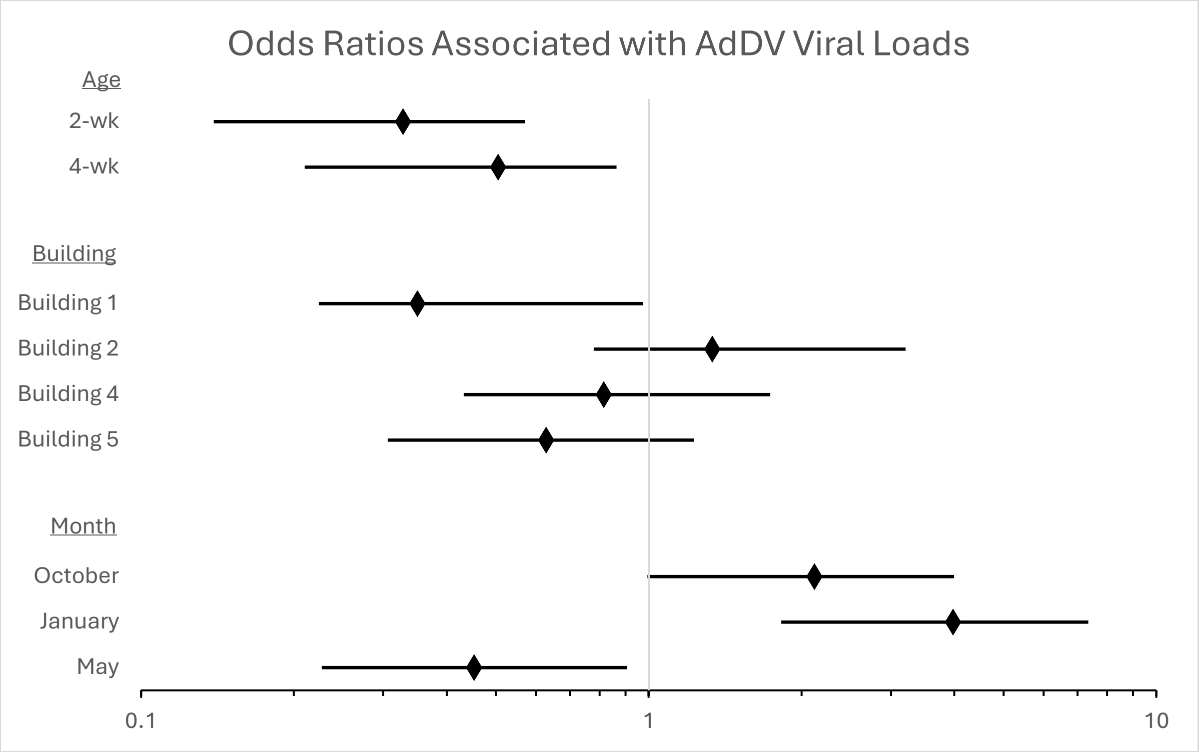

Supplementary Figure S2. This Forest plot graphically displays the odds ratios associated with AdDV viral loads and each of the risk factors described (age, building, month). For age, 2-week and 4-week-old crickets were less likely to have a high viral load as compared to the 6-week-old crickets used as reference for the model. For Building, Building 1 was found to have lower viral loads as compared to Buildings 2, 4, 5, and 6 (the reference building). For month, October and January were more likely to have higher viral loads as compared to August (the reference month) and May was less likely to have higher viral loads.

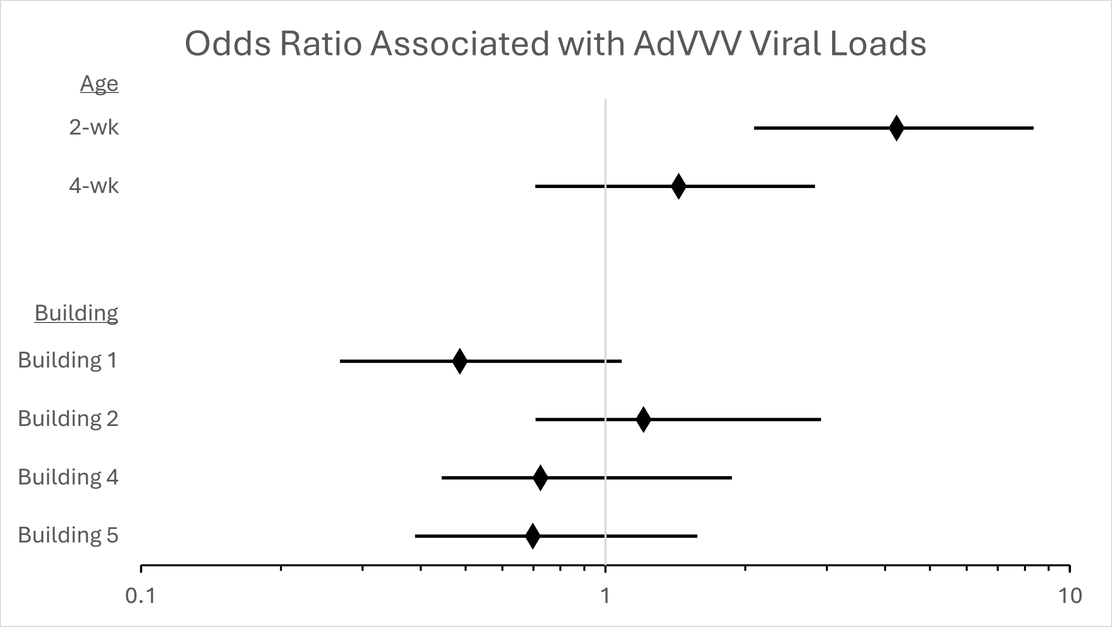

Supplementary Figure S3. This Forest plot graphically displays the odds ratios associated with AdVVV viral loads and each of the risk factors described (age and building, month did not provide reliable results). For age, 2-week were more likely to have a high viral load as compared to the 6-week-old crickets used as reference for the model. For Building, the confidence intervals crossed “1” for all buildings indicating similar results for each.

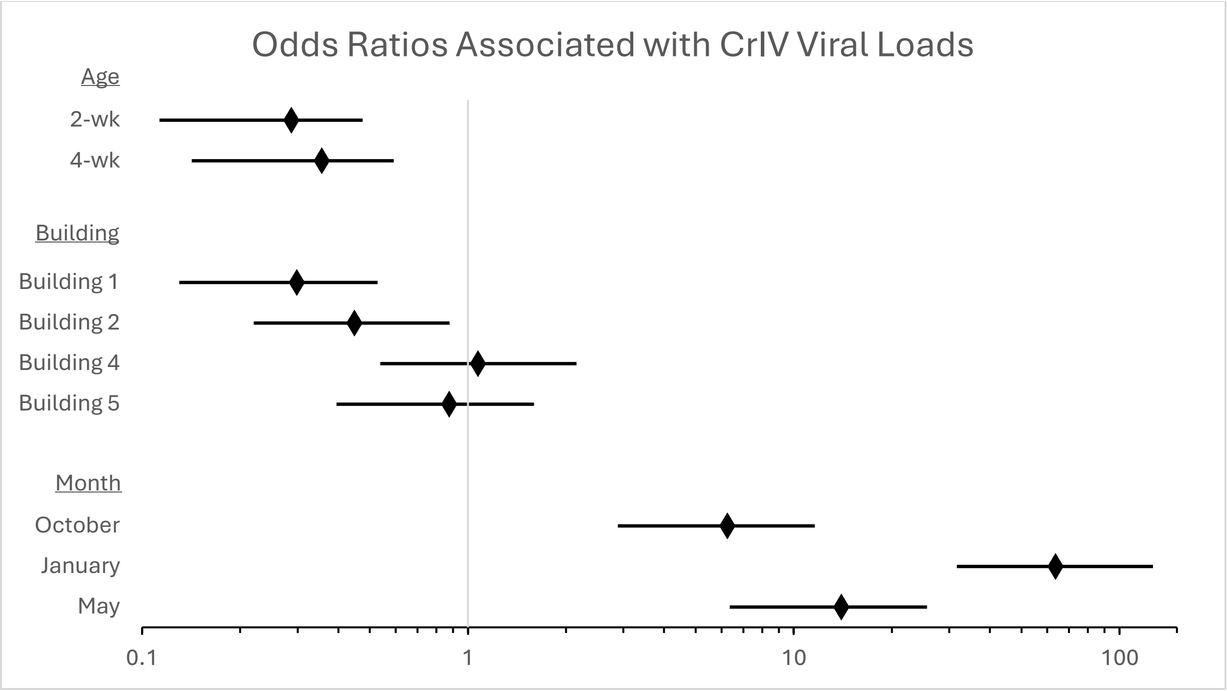

Supplementary Figure S4. This Forest plot graphically displays the odds ratios associated with CrIV viral loads and each of the risk factors described (age, building, month). For age, 2-week and 4-week-old crickets were less likely to have a high viral load as compared to the 6-week-old crickets used as reference for the model. For Building, Buildings 1 and 2 were found to have lower viral loads as compared to Buildings 4, 5, and 6 (the reference building). For month, October, January, and May were all more likely to have higher viral loads as compared to August (the reference month).
